# 儿童急性T淋巴细胞白血病微小残留病研究现状及展望

**DOI:** 10.3760/cma.j.cn121090-20240701-00239

**Published:** 2025-01

**Authors:** 凤凤 牛, 超 高

**Affiliations:** 国家儿童医学中心、首都医科大学附属北京儿童医院检验中心，儿童血液病与肿瘤分子分型北京市重点实验室，儿科重大疾病研究教育部重点实验室，北京 100045 Department of Clinical Laboratory Center, Beijing Key Laboratory of Pediatric Hematology Oncology, Key Laboratory of Major Diseases in Children Ministry of Education, Beijing Children's Hospital Capital Medical University, National Center for Children's Health, Beijing 100045, China

## Abstract

儿童急性T淋巴细胞白血病（T-ALL）因其高侵袭性和治疗复杂性而备受关注。近年来，随着医疗技术的进步和研究的深入，儿童T-ALL的治疗效果已得到显著提升。然而，微小残留病（MRD）的存在仍然是影响儿童T-ALL预后的重要因素。随着检测方法的不断发展，目前定量PCR、多参数流式细胞术和高通量测序技术的临床应用，已经能够对MRD进行精确地检测，从而实现患者个体化的精准治疗。本文旨在综述当前儿童T-ALL MRD的检测方法、多药联合化疗与造血干细胞移植中MRD监测与评估的临床意义，以及应用MRD衡量近期T-ALL中新疗法的反应性与有效性，并展望未来的研究方向和潜在的治疗策略。

## Abstract (en)

Pediatric T-cell acute lymphoblastic leukemia（T-ALL）has been attracted much attention due to its high aggressiveness and complexity of treatment. Recently, with the development of technology and clinical research, the curative effect of T-ALL in children has been significantly improved. However, the presence of minimal residual disease（MRD）is still a key factor affecting the outcomes of children with T-ALL. With the continuous development of detection methods, the clinical applications of real-time quantitative PCR, multi-parameter flow cytometry, and high-throughput sequencing technology, MRD can be detected accurately to achieve personalized and precise treatment for each patient. The purpose of this article is to review the current detection methods of MRD in T-ALL, the clinical significance of MRD monitoring and evaluation in multi-agent combined chemotherapy and hematopoietic stem cell transplantation, and applying MRD to measure the responsiveness and effectiveness of new therapies in recent T-ALLs. Research directions and potential treatment strategies in the near future were also proposed.

急性淋巴细胞白血病（ALL）是儿童期最常见的恶性肿瘤，由B或T淋巴前体细胞分化阻滞并异常增生所引起。其中，T细胞ALL（T-ALL）占新诊断儿童ALL的10％～15％[1]，通常与男性、年龄较大、高白细胞计数、纵隔肿块等临床特征相关[2]。研究显示，T-ALL与B-ALL生物学上存在很大差异，疾病反应动力学模式不同。微小残留病（MRD）是儿童T-ALL预后最重要的决定因素，当纳入MRD后，患者其他临床生物学因素以及白血病细胞的遗传学变异都不具独立预后意义[3]。MRD是患儿获得形态学缓解后体内仍残留的少量白血病细胞。MRD监测应用于初发和复发ALL，是对患者进行个体化精准治疗的基础，可指导临床危险度分层治疗的开展、异基因造血干细胞移植（allo-HSCT）前后治疗方案的调整。近年进行的分子靶向新疗法亦将MRD作为证明其有效性的评价标准。本文将聚焦目前各种儿童T-ALL MRD检测方法的优势和局限性，及其在化疗、HSCT和靶向治疗中的应用价值，以期为临床评估和治疗提供思考，改善患儿预后。

一、T-ALL MRD的检测标志和方法

MRD检测的目标是采用敏感性、特异性更好的技术方法，尽早地识别传统光镜形态学方法无法发现的白血病细胞。适宜的MRD标志需要是稳定的、ALL细胞特异性变异。此外，由于ALL细胞间存在克隆异质性，且其发生是多步骤过程，已发现儿童ALL在宫内即可存在染色体易位，而一个前白血病亚克隆细胞群获得第二事件是发展为ALL所需，因此需要将第二事件作为MRD监测的标志。第二事件是发生于ALL发育晚期的变异，可特异性存在于全部ALL细胞，或者引起复发的亚克隆细胞群中。

儿童T-ALL中常用的MRD检测技术包括多参数流式细胞术（MFC）、荧光定量聚合酶链反应（RQ-PCR）、微滴式数字PCR（ddPCR）和高通量测序技术（NGS）。T-ALL MRD检测方法的优势与局限对比详见表1。

**表1:** 急性T淋巴细胞白血病（T-ALL）微小残留病（MRD）检测方法的优势与局限性

	MFC	Ig/TR RQ-PCR	融合基因RQ-PCR	Ig/TR ddPCR	Ig/TR NGS
覆盖患者比例	>95％	90％~95％	15％~35％	90％~95％	95％
灵敏度	10^−4^~10^−5^	10^−4^~10^−5^	10^−4^~10^−6^	10^−4^~10^−6^	10^−6^~10^−7^
优势	1. 快速且相对便宜，应用广泛 2. 可绝对定量MRD 3. 提供细胞组成信息 4. 分析中排除死细胞	1. 灵敏度、特异性高 2. 可以在不同样本之间进行比较 3. 患者的重排位点是特异的，不存在PCR后的操作过程，交叉污染低风险 4. 技术成熟，欧洲MRD联盟已建立细致的标准，广泛应用	1. 监测标志在疾病和治疗过程中稳定 2. 不需构建患者特异性流程，操作简便 3. 应用广泛，欧洲MRD联盟已建立部分标志的标准化流程	1. 灵敏度高，且能提供靶DNA样品的绝对定量 2. 数据分析相对简单 3. 不需消耗初诊DNA样本	1. 灵敏度、特异性及适用性更高 2. 欧洲克隆性NGS联盟已建立标准化流程 3. 全部患者都使用通用引物组合 4. 可识别和监测克隆演化标志 5. 不需消耗初诊DNA样本
局限性	1. 对样本质量和操作人员技术要求高 2. 标准化程度较低 3. 每个患者最好应用2个以上异常表达免疫表型标志，避免免疫表型漂移可能导致的假阴性结果 4.药物诱导可能影响抗原表达水平，导致假阴性结果	1. 可能存在引物设计问题、DNA质量问题、PCR条件设置问题，影响检测结果的准确性 2. Ig/TCR基因重排可能发生克隆演化，将造成假阴性结果 3. 存在生理背景的非特异扩增；低水平MRD时不易区分 4. 低活力或者死亡细胞的DNA也可被扩增 5. 患者特异性引物设计及其反应条件优化耗时较多 6. 后续追踪过程，需消耗初诊DNA建立标准曲线	1. 未检出融合基因的T-ALL无法适用 2. 非患者特异性，可能会有患者间污染 3. RNA不稳定，易降解 4. 低活力或者死亡细胞的DNA也可被扩增	1. 费用相对较高，未标准化 2. 依赖于等位基因特异性引物 3. 未建立数据分析指南 4. 仅部分实验室应用范围有限，需进一步探索和验证	1. 检测成本高，时间长 2. 生物信息学分析仍具有挑战性 3. 实验室间未标准化 4. 如需达到高敏感度定量目标值，需要大骨髓样本 5. 低活力或者死亡细胞的DNA也可被扩增

**注** MFC：多参数流式细胞术；Ig/TR：免疫球蛋白/T细胞受体；RQ-PCR：荧光定量聚合酶链反应；ddPCR：微滴式数字PCR；NGS：高通量测序技术

MFC通过识别诊断时白血病相关抗原，以及系别和（或）发育阶段的差异表达抗原，与正常淋巴细胞进行区分，并进行后续MRD评估。由于正常的T细胞前体在胸腺中并不进入循环，因此应用T-ALL前体细胞的特征足够来鉴定骨髓或者外周血中的T-ALL细胞。T-ALL典型的MFC组合中包含白血病细胞不同步表达的成熟过程相关抗原CD2、CD3、CD5、CD7、CD8、CD34和CD45，同时也可检测T-ALL中异位表达的胸腺抗原。美国儿童肿瘤协作组（COG）检测T-ALL的免疫MRD组合见表2，并且通过应用核酸结合染料直接计数有核细胞作为分母管对结果进行归一化[4]–[6]。意大利儿童血液肿瘤协作组-柏林-法兰克福-明斯特（AIEOP-BFM）ALL流式研究组报道儿童T-ALL中，CD48和CD99是监测MRD非常有益的标志[6]。近年来，基于6～8色流式细胞术的MRD检测敏感度得到大幅提升，敏感度可达10^−4^～10^−5^，覆盖99％以上的患者[7]。但该方法对检测者的技术和经验要求较高，且易受检测仪器性能的影响，因此需要建立严格的质量控制体系。

**表2:** 美国儿童肿瘤协作组急性T淋巴细胞白血病微小残留病检测免疫分型组合

	PB	FITC	PE	PE-TR	PC5	PC7	A594	APC	APC7
管1	CD16	cCD3	CD7	–	CD56	CD5	CD38	sCD3	CD45
管2	CD8	CD48	CD5	CD34	CD56+16	CD3	CD4	CD7	CD45

**注** 该组合需4激光流式细胞仪，包括407、488、594和635 nm

克隆性免疫球蛋白/T细胞受体（Ig/TR）基因重排是T-ALL常见的MRD检测分子标志物，T-ALL中可有20％的患者存在跨系表达的Ig基因重排[8]。Ig/TR基因重排可见于约95％的患者，且为患者特异性标志，但该标志不直接与白血病的发生相关，且在疾病过程中可经V（D）J重组酶介导出现继发性重排或演化，因此在MRD检测中最好检测2个以上单克隆标志以避免假阴性结果。在T-ALL中，Ig/TR重排相对稳定，约90％在复发时仍然保留[9]。EuroMRD工作组建立了RQ-PCR方法检测Ig/TR重排的标准化方案[10]，该方法需要合成等位基因特异性引物，优化每一个反应，并需要在后续MRD检测中应用初诊DNA构建标准品。近年来，EuroClonality-NGS联盟建立了以Ig/TR重排为标志的NGS-MRD检测方法[11]，能够提供更加全面的定量信息，评估克隆大小，特别适用于寡克隆和双克隆重排。如果DNA样本量足够，该方法敏感度可以达10^−7^。该方法在精确定量MRD的同时，还能提供正常免疫组库的信息。在诱导后或HSCT后监测MRD，NGS对预测复发比RQ-PCR方法更加特异，并且更易发现克隆演化[12]–[13]。需要注意的是，目前NGS-MRD检测方法仍需标准化，尚未在临床广泛应用。

白血病特异性融合转录本也可作为T-ALL MRD检测的标志，但该标志非患者特异性，因此容易出现实验室污染和假阳性结果，且并非每例患者都可检测出。欧洲抗击癌症计划（Europe Against Cancer Program）已建立了RQ-PCR检测T-ALL SIL-TAL1融合转录本的标准化定量方案[14]，但由于不同患者融合转录本表达水平差异极大，且RNA降解速率不同，因此需对检测结果进行归一化处理[15]。既往研究利用PCR方法，针对DNA水平的SIL-TAL1融合进行MRD检测，敏感度可达10^−5^［16]。

ddPCR是较新的MRD监测技术。该方法将反应体系分割为大约20 000个小液滴，每一个DNA片段在其中分别扩增，因此该方法作为绝对定量不需建立标准曲线。ddPCR方法的敏感度、准确性和可重复性优于RQ-PCR方法，更加适用于低浓度靶标，但是目前该方法尚未标准化，仍有待临床进一步验证。

T-ALL中独特的早期前体T细胞（Early T-cell Precursor，ETP）表现为不成熟的T系免疫表型，具有髓系抗原表达，而大多数ETP-ALL细胞未进行Ig/TR重排或者表现为寡克隆性，因此影响了这一MRD监测标志的应用。而髓系的FLT3-ITD标志，在ETP-ALL中存在率较高，可作为MRD追踪标志[17]。

临床上，通常应用骨髓样本进行MRD评估。鉴于外周血样本更易获得，且操作侵入性低于骨髓穿刺，近年来一些研究尝试利用外周血替代骨髓进行MRD检测。儿童和成人T-ALL的临床研究均表明，外周血与骨髓MRD检测水平有很强的相关性，二者MRD水平类似[18]，提示我们可应用侵入性更低的外周血样本替代骨髓来完成T-ALL治疗过程中频繁的MRD评估。此外，一项通过流式细胞术进行MRD评估的成人ALL研究显示，仅有的2例外周血MRD阳性而骨髓MRD阴性患者均为T-ALL[19]，提示对于胸腺起源的T-ALL，在外周循环中检测到白血病细胞的倾向性高于骨髓，外周血MRD检测是骨髓MRD检测的有益补充，利于髓外复发监测和尽早进行治疗干预。

二、T-ALL一线治疗中MRD监测的临床意义

1. 治疗早期MRD评估在患者预后判断中的意义：MRD综合反映了患者体内白血病细胞、宿主和治疗方法等的药物抵抗，是独立预后因子，不同基因和生物学亚型患者治疗早期MRD不同，此外MRD对于相同亚型的ALL患者也具有预后价值。初诊儿童T-ALL中，MRD的预后意义高于患者其他的临床和生物学因素，当纳入MRD进行预后分析，MRD是唯一的独立预后因素，而其他变量都不具独立预后意义，T-ALL的MRD可以预测全身和髓外复发[20]。T-ALL患者早期清除MRD的速度与预后相关。美国St. Jude XIII研究通过RQ-PCR监测MRD，第19天MRD<10^−4^者，5年累计复发率（CIR）较低，而其ⅩⅤ 研究显示，第19天MRD≥1％者预后较差，即使诱导治疗结束时（End of induction, EOI）MRD阴性，预后也差于其他亚型[21]。全球首个大样本多中心MRD指导的随机临床研究AIEOP-BFM-ALL 2000中，第15天MFC和（或）Ig/TCR MRD<0.1％者，CIR仅为3.3％，而≥10％者高达55.6％[22]；EOI-MRD <0.01％者，5年无事件生存（EFS）率较高[23]；但同时EOI-MRD低水平（0.001％～0.01％）阳性的患者复发风险增高[24]。该研究也表明，儿童T-ALL MRD清除较慢，约84％儿童T-ALL患者EOI-MRD为阳性，巩固治疗结束时（End of consolidation, EOC）MRD阴性者，经传统化疗7年EFS率尚可；而EOC-MRD≥10^−3^的患者复发率较高，预后不良[23]。中国儿童白血病协作组（CCLG）ALL-2008多中心临床研究中通过MFC检测的96例儿童T-ALL MRD也证实，EOC-MRD最具预后价值，≥10^−2^者5年EFS率仅12.5％[25]。而美国COG AALL0434临床研究采用MFC评估MRD，对儿童和青少年T-ALL分析显示，EOI-MRD≥1％和EOC-MRD≥0.1％均被证实是不良预后因素；而在近，ETP-ALL和非ETP-ALL患者中EOI-MRD>0.1％者预后差[5]。

2. MRD指导的危险度分层和治疗策略的调整：T-ALL的预后决定因素是EOI和EOC的MRD水平，在危险度分层中与B-ALL不同，并未纳入白血病细胞生物学特征。MRD高低依赖于患者的治疗方案以及样本采集时间，此外一个方案的MRD分层标准，不能简单外推到其他方案，而需要先在相同治疗情况下回顾性分析MRD的临床影响。由于MRD监测技术方法、治疗方案设计不同，因此不同白血病临床研究组应用单一或多个MRD评估时间点，以及不同的MRD分界值来进行危险度划分。AIEOP-BFM ALL 2000 EOI和EOC-MRD均<10^−4^者为标危，EOC-MRD≥10^−3^（后期改为≥5×10^−4^）为高危，其余为中危[26]。COG根据MRD水平将T-ALL分为三个危险度，第29天MRD<0.01％的患者进入标危组，第29天MRD≥0.01％ EOC MRD<0.1％为中危组，EOC-MRD≥0.1％为高危组。我国CCLG-ALL 2018中根据MRD水平区分T-ALL患儿的危险度，高危患者的MRD标准是第15天≥10^−1^或EOI ≥10^−2^或巩固治疗前≥10^−4^。

T-ALL不常规在第1次完全缓解（CR）后进行HSCT，除非后期MRD持续阳性。我国CCLG-ALL 2018方案推荐T-ALL在早期强化CAML1方案结束后骨髓评估MRD≥10^−2^以及高危患儿在HR-1′方案治疗后至HR-2′治疗前MRD≥10^−4^者进行HSCT治疗。AIEOP-BFM ALL 2000临床研究，对第78天MRD≥10^−3^者升级至高危组，并在后续每一个再巩固单元结束时进行MRD评估，以调整治疗强度为进行HSCT做准备[23]。COG HSCT的指征包括第29天MRD>0.1％但巩固后形态学为M2或M3者，以及第43天MRD>1％的CR_1_患者[27]。美国St. Jude儿童医院ⅩⅤ 临床研究是首个诱导缓解后连续定期进行T-ALL外周血MRD监测并指导治疗的前瞻性临床研究中，诱导后MRD持续阳性或者出现水平升高者是进行HSCT的指征[28]。我国一项纳入136例儿童T-ALL的研究发现，在以MRD分层为指导进行HSCT后可以改善患儿预后[2]。

MRD监测常规应用于治疗过程中和治疗结束后，NCCN指南建议每3～6个月进行MRD检测至少5年。规律监测MRD，不仅可以早期发现具有复发风险的患者，及时进行治疗干预，MRD持续阴性的患者亦可避免进行HSCT及其相关死亡。此外，MRD评估亦作为新药应用的指标。COG针对初发高危T-ALL患者，将奈拉滨纳入诱导后MRD>1％患者的治疗中，其预后与MRD早期反应迅速者相当[29]，避免了应用HSCT。目前，欧洲将奈拉滨用于治疗早期MRD≥5％的初诊T-ALL患者和复发患者。日本ALL-T11全国多中心临床研究，应用奈拉滨治疗EOC-MRD高危和极高危患者[30]。

白血病治疗中面临的一个最具挑战性的目标是在治愈患者的同时，降低强化疗引起的不良反应。然而，减低强度要求MRD检测敏感度高，避免假阴性结果。在UKALL2003方案中，T-ALL患者EOI-MRD<10^−4^者，将接受标准BFM巩固方案，以及应用门冬酰胺酶替代剂量递增甲氨蝶呤，患者5年EFS率可达93％[31]。UKALL2003和FRALLE2000T通过Ig/TR监测MRD，将MRD<10^−4^与基因突变相结合，可以发现预后良好的低风险儿童T-ALL患者进行降级治疗[32]。

三、HSCT前后MRD评估的临床意义

MRD评估不仅可以识别需要进行HSCT的患者，对HSCT治疗的患者同样具有预后意义。HSCT前MRD水平影响患者移植后的预后，MRD阳性与移植后复发和不良预后相关。捷克儿童血液治疗组研究显示，5例HSCT前MRD阴性T-ALL均未复发，而1例阳性者出现复发[33]。纳入102例成人T-ALL的研究发现移植时MRD阳性患者的进展率明显高于MRD阴性患者（76％对34％）。更多的临床研究虽未单独对T-ALL进行阐述，但均提示HSCT前MRD对移植后的预后具有显著影响[34]–[36]。此外，在一项应用ALLR3和ALL-REZ BFM 2002进行再诱导治疗的复发儿童ALL研究中，T-ALL患者EOI-MRD反应良好与不良者的DFS、OS及CIR差异均具有统计学意义，但移植前MRD≥10^−3^不是DFS和OS独立的不良预后因素，提示低MRD水平的T-ALL患者可能还存在髓外白血病，而骨髓MRD检测未能发现[37]。

法国的一项儿童ALL临床研究也显示，HSCT前后MRD都与患者预后相关，且HSCT+90 d MRD还指导治疗干预[38]–[39]。移植后监测MRD是复发强有力的预测因子[39]，其敏感度和特异性均优于嵌合度分析[40]。HSCT后的儿童ALL通过连续监测MRD进行治疗方案的调整预防复发。以上两项研究中均包含儿童T-ALL患者，虽未单独说明T-ALL患者的情况，但患者与整体保持一致。此外，有成人ALL的研究表明，HSCT后MRD预测复发比HSCT前MRD更准确，其监测与临床的相关性更强[41]。因此，亟须在HSCT前后应用新疗法获得或维持MRD阴性。

由于HSCT后免疫重建，正常再生细胞和ALL幼稚细胞可以具有相似的免疫表型，降低了MRD评估的准确性和特异性。此外，HSCT后MRD对白血病细胞清除动力学的预测性减低，使临床解读变得复杂。由于免疫抑制剂的撤退及进行供者淋巴细胞回输（DLI）可能会引起严重的急慢性移植物抗宿主病（GVHD），所以只有当MRD≥10^−4^时才调整治疗。

四、MRD评估新疗法的反应性及其作为替代指标评估新疗法有效性

近年来，随着新的方法和手段应用于ALL的治疗，越来越多的研究将MRD用于评估患者对新疗法的反应性，以及新药批准适应证。我国研究团队采用NSCD7 CAR-T细胞治疗复发/难治性儿童和成人T-ALL/LBL，在输注后第28天进行MRD检测，19例患者均获得MRD阴性，除1例患者在后续HSCT治疗中死于GVHD，其余18例患者均维持CR状态；而1例第28天MRD未获得CR患者，于第43天复发，第83天死亡[42]。而应用米托蒽醌与伊达比星治疗儿童复发ALL的一项研究显示，MRD并不能预测治疗效果，说明MRD错误地评估了药物效果，不能将反应性等同为疗效[43]。尽管MRD作为ALL EFS强有力的预测因子已常规应用于治疗反应评估和分层治疗方案，但能否将早期MRD反应作为评估治疗干预EFS有效性的替代指标，以及在早期治疗阶段对新药应用的反应性评估来代替传统的终点事件仍需谨慎。后续治疗的复杂性及强度，可能会影响MRD的替代性能。因此在多药治疗背景下，评估随机治疗干预对长期EFS的影响中，需要谨慎应用早期MRD作为代替指标。MRD作为早期治疗反应的指标，能够转化为长期疗效的替代指标，依赖于后续治疗能否将良好反应长期转化。

五、总结与展望

MRD评估是儿童T-ALL预后判断和指导治疗决策的重要工具。临床常用的MRD检测技术包括MFC、RQ-PCR、NGS。近年来MFC、RQ-PCR技术的敏感度有所提升，可达10^−4^～10^−5^，NGS技术对白血病克隆的检测敏感度更高，未来有望替代RQ-PCR技术，但仍需标准化。随着科技的飞速发展和医学研究的不断深入，MRD检测技术也将迎来更新的突破。整合应用基因组学、转录组学、表观遗传组学、蛋白质组学和代谢组学等的多组学技术，以及血液病学、肿瘤学、分子生物学、遗传学、生物信息学等多学科合作模式，也必将应用到ALL-MRD监测领域中。尽管既往采用骨髓样本进行MRD检测，但在T-ALL中外周血和骨髓样本检测结果的一致性很好，提示T-ALL中应用更易获得的外周血样本，特别是开展液体活检技术，监测cfDNA和ctDNA进行MRD评估的可能性和广阔前景。

实验室间对MRD的判定差异和不同临床试验采用不同的MRD评估技术，给临床试验结果间的比较造成困难。尽管如此，既往研究普遍证实了治疗早期MRD在T-ALL患者预后判断、危险度划分、治疗方案调整等方面的重要作用。近年来，随着T-ALL基因组学研究进展，不仅能够提升精细化分层分型治疗和靶向治疗的开展，未来MRD评估也将迎来患者个体化监测方案；基础研究对T-ALL，特别是耐药的MRD白血病细胞生物学特性的揭示，有助于新治疗策略及治疗靶点的开发。同时，MRD能否作为新药评估终点的替代指标，以及基于MRD的分子复发能否替代现有复发定义也将更加明确。MRD指导的前瞻性临床研究，也将使患者进一步获益，预防复发、改善预后。
